# Metagenomic Analysis of the Species Composition and Seasonal Distribution of Marine Dinoflagellate Communities in Four Korean Coastal Regions

**DOI:** 10.3390/microorganisms10071459

**Published:** 2022-07-19

**Authors:** Jinik Hwang, Hee Woong Kang, Seung Joo Moon, Jun-Ho Hyung, Eun Sun Lee, Jaeyeon Park

**Affiliations:** 1 West Sea Fisheries Research Institute, National Institute of Fisheries Science, Incheon 22383, Korea; jinike12@korea.kr (J.H.); hwgang@korea.kr (H.W.K.); 2 Environment and Resource Convergence Center, Advanced Institute of Convergence Technology, Suwon 16229, Korea; sjmoon04@snu.ac.kr (S.J.M.); hjh1120@snu.ac.kr (J.-H.H.); eunsun742@snu.ac.kr (E.S.L.)

**Keywords:** dinoflagellates, metagenomics, next-generation sequencing, monitoring

## Abstract

Biomonitoring of dinoflagellate communities in marine ecosystems is essential for efficient water quality management and limiting ecosystem disturbances. Current identification and monitoring of toxic dinoflagellates, which cause harmful algal blooms, primarily involves light or scanning electron microscopy; however, these techniques are limited in their ability to monitor dinoflagellates and plankton, leaving an incomplete analysis. In this study, we analyzed the species composition and seasonal distribution of the dinoflagellate communities in four Korean coastal regions using 18S rRNA amplicon sequencing. The results showed significantly high diversity in the dinoflagellate communities in all regions and seasons. Furthermore, we found seasonally dominant species and causative species of harmful algal blooms (*Cochlodinium* sp., *Alexandrium* sp., *Dinophysis* sp., and *Gymnodinium* sp.). Moreover, dominant species were classified by region and season according to the difference in geographical and environmental parameters. The molecular analysis of the dinoflagellate community based on metagenomics revealed more diverse species compositions that could not be identified by microscopy and revealed potentially harmful or recently introduced dinoflagellate species. In conclusion, metagenomic analysis of dinoflagellate communities was more precise and obtained results faster than microscopic analysis, and could improve the existing monitoring techniques for community analysis.

## 1. Introduction

Marine dinoflagellates are ubiquitous and play diverse roles in marine ecosystems [1,2]. Some dinoflagellate species can grow out of control due to various environmental factors, such as excessive inorganic nutrients (nitrogen (N) and phosphorus (P)) introduced from the land, forming a bloom [3,4]. Blooms from dinoflagellates have detrimental effects on a variety of aquatic animals, including fish and aquatic mammals, and can even be harmful to humans through toxin production [5,6]. Therefore, continuous monitoring of dinoflagellate communities is essential, as they can affect the diversity of surrounding aquatic life and cause ecosystem disturbance.

To date, monitoring of dinoflagellates in the aquatic environment has generally involved morphological identification using light microscopy observations. Recent advances in microscopy, including scanning electron microscopy (SEM), have enabled more precise identification [7,8]. However, the morphological classification of plankton via microscopy is still challenging, as plankton are difficult to observe with SEM due to the lack of an outer shell in dinoflagellates or the extremely small size of plankton. Recently, many types of species identification technology to distinguish dinoflagellates and molecular technology targeting species-specific genes have been developed [9,10]. In particular, next-generation sequencing (NGS) has greatly expanded our understanding of the diversity and function of dinoflagellates in the aquatic environment. This technique allows for rapid, high-resolution analysis of microbial and dinoflagellate communities [11,12]. In addition, it is possible to accurately identify nano- and pico-sized plankton, which are difficult to distinguish with a conventional microscope, facilitating the identification of various plankton that have been overlooked because they do not appear or are difficult to distinguish in local environmental conditions [13]. Although the QIIME or USEARCH pipeline has been widely used to analyze 16S rRNA gene sequencing reads from microbial communities [14,15,16], many metagenomics studies examining the profile of marine dinoflagellates have been carried out using the CLC Genomics Workbench [17,18,19,20]. In this study, we analyzed taxonomic profiling and seasonal distribution of the dinoflagellate communities in four Korean coastal regions based on the reading of 18S rRNA sequences using the CLC Workbench. To verify the results calculated using the CLC tool, those results were compared with abundance measured by direct counting of cells using microscopy.

Outbreaks of harmful dinoflagellates have traditionally occurred in tropical or temperate regions which have the potential for enhancing the growth rate of phytoplankton cells under the appropriate environmental conditions. Jeju Island, located along the southern coast of Korea, is a temperate region, and the occurrence of benthic dinoflagellates producing phytotoxins has been frequently reported in Jeju [21]. Understanding the spatial and seasonal dynamics of the toxic dinoflagellates in this region is essential, and many researchers have continuously monitored the cell abundance around Jeju Island using microscopic identification [22,23,24]. In this study, we investigated the spatial and seasonal variation of dinoflagellate communities in four different sites in Korean coastal waters, including Jeju Island, using NGS-based (18S rRNA amplicon) metagenomics. For precise bioinformatics analyses, we established a reference database of dinoflagellates and analyzed the precision of NGS compared to conventional microscopic observation. Thus, the reference data for the dinoflagellate community classified based on the NGS findings in this study will provide a better understanding of the occurrence of toxic dinoflagellates in Korea.

## 2. Materials and Methods

### 2.1. Study Areas and Seawater Sample Collecting

**Figure 1:**
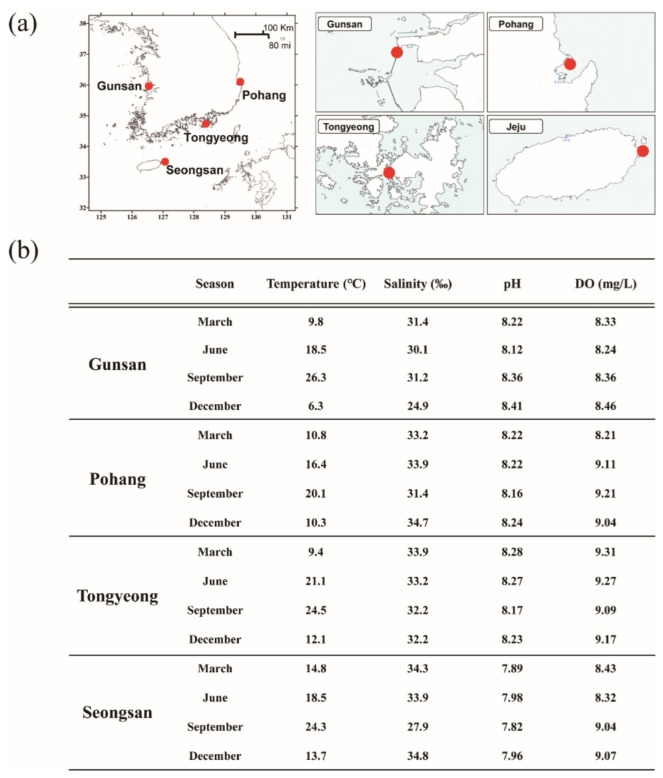
Location of sample sites and environmental indices at these sites (**a**) in four regions of Korean coastal waters (**b**).

Seawater samples for metagenomic analysis were collected from four coastal waters (Gunsan, Pohang, Tongyeong, and Seongsan) in March, June, September, and December 2019. The four selected sampling sites have different geological and environmental characteristics, representing the eastern coast (Pohang), southern coast (Tongyeong), western coast (Gunsan), and Jeju island (Seongsan), and all four locations are near a port with considerable human activity (Figure 1a). To remove large zooplankton and foreign substances in the sample, surface seawater at each region was sieved using meshes with pore sizes of 80 µm. Four liters of seawater samples for metagenomics analysis were filtered through a polycarbonate filter membrane (0.8 µm Millipore; MilliporeSigma, Burlington, MA, USA) to obtain environmental DNA samples, then transferred to the laboratory on dry ice. For microscopic analysis, 500 mL of seawater samples was fixed with Lugol’s solution, and phytoplankton cells were identified to at least the genus level using an optical microscope (Axioskop; Zeiss, Oberkochen, Germany). The dinoflagellate cells were counted directly using a Sedgwick-Rafter counting chamber by light microscopy (BX53; Olympus, Tokyo, Japan). Environmental data, such as water temperature, pH, dissolved oxygen, and conductivity, were measured at each location using a YSI 566 Multi Probe System (YSI Inc., Yellow Springs, OH, USA).

### 2.2. DNA Extraction, Library Preparation, and NGS

DNA was extracted from the filtered membranes containing dinoflagellates and microbial cells using a DNeasy PowerSoil Kit (Qiagen, Hilden, Germany) following the manufacturer’s instructions. The amount of double-stranded DNA and the purity in the extracted DNA samples was measured by PicoGreen (Promega, Madison, WI, USA) using VICTOR Nivo (PerkinElmer, Waltham, MA, USA). Per the Illumina 16S Metagenomic Sequencing Library protocols, the V3-V4 region of 18S ribosomal DNA (rDNA) gene in each sample was amplified by PCR using the following primers: 18S amplicon PCR forward primer, 5′–TCGTCGGCAGCGTCAGATGTGTATAAGAGACAGCCAGCASCYGC GGTAATTCC-3′, reverse primer, 5′–GTCTCGTGGGCTCGGAGATGTGTATAAG -AGACAGACTTTCGTTCTTGATYRA-3′ [25]. A subsequent amplification step with limited-cycle reaction was performed to add multiplexing indices and Illumina sequencing adapters. The PCR products were pooled, cleaned, and normalized using the PicoGreen, and the size of libraries was measured using a TapeStation DNA screen tape D1000 (Agilent Technologies, Santa Clara, CA, USA). Sequence libraries in the sample were verified using the MiSeq™ platform (Illumina, San Diego, CA, USA).

### 2.3. Customized Dinoflagellate Reference Databases for CLC Workflows

For the DNA reference databases of dinoflagellates, a list of 1555 species of dinoflagellates named in a previous study [26] was prepared in the form of Excel data, and the reference database deposited in the NCBI was additionally downloaded. A total of approximately 5000 dinoflagellate reference databases were retrieved. The files were imported into CLC and customized for use as databases specified for analyzing dinoflagellate species. The analysis program used in this study was CLC Genomics Workbench 21.0.4 with CLC Microbial Genomics Module 21.0 (CLC Bio, Qiagen Company, Aarhus, Denmark) and was used for future species identification (Figure S1).

### 2.4. Data Quality Control and Taxonomic Profiling

Data quality control and taxonomic profiling were performed using the CLC Microbial Genomics Module (MGM). First, Reads were trimmed using the Trim Reads tool. The percentage of trimmed from approximately 300,000 reads per sample was 71% (*n* = 16). We trimmed the 5′ and 3′ terminal nucleotides of the reads, and discarded unqualified reads showing that the quality limit was less than 0.001 or ambiguous nucleotides were more than two. The average length of reads after trimming was between 217–234 bp. Samples with less than 100 reads (minimum percent from the median = 50.0) were removed. Second, the remaining qualified reads were used for operational taxonomic unit (OTU) clustering based on SILVA 18s v132 Database including 1555 dinoflagellates at a 97% sequence similarity. The detected chimeric sequences and singletons (Chimera crossover cost = 3, K-mer size = 6) were discarded. A phylogenetic tree using the neighbor-joining method with 100 replicates was constructed based on the aligned OTU sequences by the MUSCLE tool v3.8.425. The phylogeny was applied for alpha and beta diversity measures. The beta diversity was measured using the Euclidean distance, and principal coordinate analysis (PCoA) based on a Bray–Curtis dissimilarity matrix was performed to illustrate a hierarchical clustering heat map showing the correlation between the examined samples.

## 3. Results

### 3.1. Environmental Characteristics of Sampling Sites

The four selected sampling sites had different geological and environmental characteristics. All the regions showed four distinct seasons; however, there was a regional difference in water temperature. The month of March showed the lowest water temperature (6.4–14.3 °C) throughout the region, and September (20.1–26.3 °C) showed the highest water temperature. On average, the water temperature at Jeju Island (Seongsan) was higher than that of the land. The salinity did not show a significant difference by region (31.4–33.7‰), and the pH and dissolved oxygen amount also did not show significant regional changes (Figure 1b).

### 3.2. Metagenome Comparisons

**Figure 2:**
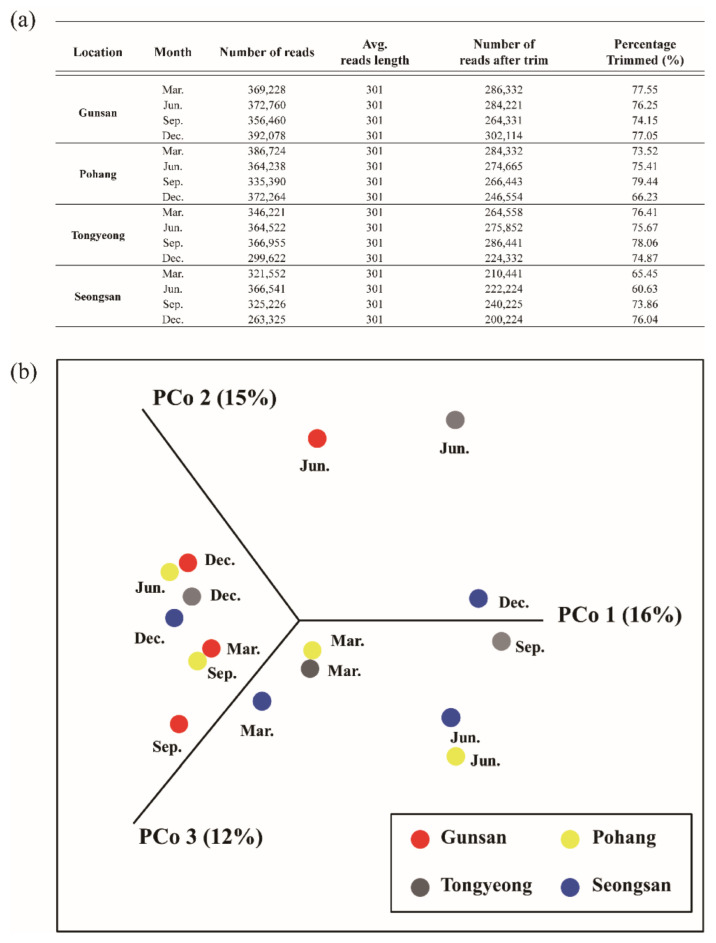
Comparison of metagenome libraries. Next-generation sequencing metadata including number of reads and trimmed reads (**a**), β-diversity (principal coordinate analysis (PCoA), dinoflagellate genotype composition (proportions) was measured by Bray–Curtis distances (**b**).

A pipeline for metagenomics analysis of environmental DNA samples was developed to address the identification of dinoflagellates species. On average, over 300,000 reads were acquired from each region using the MiSeq™ platform (Illumina, San Diego, CA, USA), with a read length of 301 bp. After quality trimming and filtering of reads, 70.3% of the raw reads remained (Figure 2a), with an overall higher G+C content for reads obtained from the library.

The nucleotide sequence similarity of the dinoflagellate genes was expressed by region using PCoA to illustrate the overall regional similarity according to the season. The December samples for Gunsan, Tongyeong, and Seongsan showed similarities, and the March samples of Pohang, Tongyeong, and Seongsan were also similar. The June and September samples of Tongyeong, in which a single species bloomed and became dominant, showed no similarity with the other samples. Furthermore, low similarity was found at Gunsan in June compared with the other samples (Figure 2b).

### 3.3. Metagenomic Analysis of the Dinoflagellate Species Composition

To identify marine dinoflagellates, we used the CLC genomics workbench program (CLC Microbial Genomics Module) on the assembled read sequences, followed by BLAST searches on the NCBI database and the newly created database of 1555 dinoflagellate species. Following the metagenomic analysis, 64 species of dinoflagellate were found in all regions on average. The top 10 dinoflagellates were selected based on the analyzed reads (Table 1).

**Figure 3:**
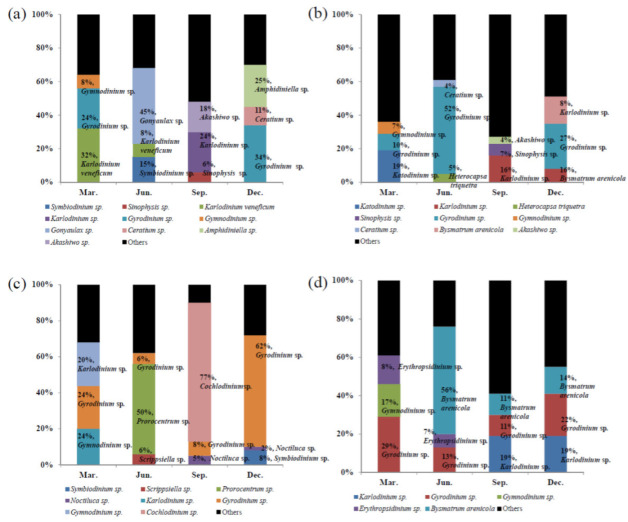
Proportion of top 3 most abundant species in each coastal seawater by metagenome analysis. Gunsan (**a**), Pohang (**b**), Tongyeong (**c**), Seongsan (**d**).

In Gunsan, the western coast, the highest number of reads detected by metagenomics data was seen in December (Table 1a). In March, two species (*Karlodinium veneficum* and *Gyrodinium* sp.) were dominant. When the ratio (%) of the top 10 species was calculated based on the total reads matched with dinoflagellate sequence, *Karlodinium veneficum* was the most dominant species, approximately 32%. Next, *Gyrodinium* sp. (24%) and *Gymnodinium* sp. (8%). In June, the composition of *Gonyaulax* sp. showed approximately 45%, followed by that of *Symbiodinium* sp. (15%) and *Karlodinium veneficum* (8%). Similar to March, the dominant species in September was *Karlodinium* sp., which accounted for 24%. In December, *Gyrodinium* sp. (34%) and *Amphidiniella* sp. (25%) were dominant as well as *Ceratium* sp. which accounted for 11% (Table 1b, Figure 3a).

In Pohang, the eastern coast, *Gyrodinium* sp. was dominant in June and December, *Katodinium* sp. was dominant in March, and *Karlodinium* sp. was dominant in September. (Table 1, Figure 3b). In Tongyeong, the southern coast, the appearance of *Gyrodinium* sp. was high in March and December, and was dominant at 24% and 62%, respectively. In particular, *Cochlodinium* sp. formed a red tide and dominated over 77%, and in June, the dominance of *Prorocentrum* sp. was more than 50%. The diversity was the highest in December, when 28 species of dinoflagellate reads were detected (Table 1, Figure 3c). In Seongsan, *Gyrodinium* sp. appeared at a high rate in all seasons, while *Bysmatrum arenicola* and *Karlodinium veneficum* dominated in June (56%) and September (19%), respectively. The sand-dwelling dinoflagellate *Bysmatrum arenicola* was dominant at Seongsan, except in March (Table 1, Figure 3d).

**Figure 4:**
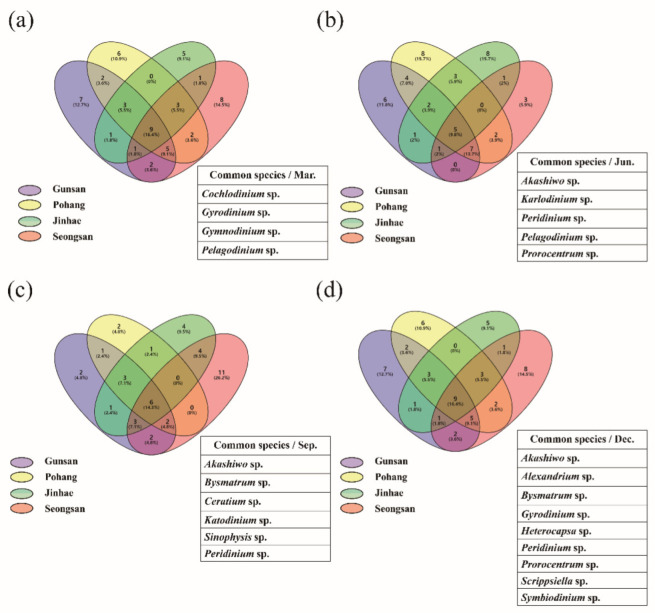
Seasonal common dinoflagellate species in 4 coastal waters by metagenome analysis. March (**a**), June (**b**), September (**c**), and December (**d**).

Figure 4 illustrates the most common species in the four coastal waters. In March, there were three common species at all sampling sites: *Cochlodinium* sp., *Gyrodinium* sp. *Gymnodinium* sp., and *Pelagodinium* sp. The most common species in June were *Akashiwo* sp., *Karlodinium* sp., *Peridinium* sp., *Pelagodinium* sp., and *Prorocentrum* sp. In September, *Akashiwo* sp., *Bysmatrum* sp., *Ceratium* sp., *Katodinium* sp., *Sinophysis* sp., and *Peridinium* sp. were common. The common species in December were *Akashiwo* sp., *Alexandrium* sp., *Bysmatrum* sp., *Gyrodinium* sp., Hetrocapsa sp., *Peridiniopsis* sp., *Prorocentrum* sp., *Scrippsiella* sp., and *Symbiodinium* sp.

### 3.4. Comparison of Metagenomic Analysis and Microscopic Observation

When the abundance of dinoflagellates was analyzed by microscopic observation, the number of species composition was mostly lower than from metagenomic analysis (Table 2). Overall, the number of species in December was lower than in other seasons, as the biomass was considerably low and mainly dominated by diatoms. At Gunsan, the abundance of *Gyrodinium* sp. species was 0.8–2.9 cells mL^−1^ in March, September, and December, which showed similar patterns to the metagenomic analysis. In Pohang, the species composition in June was more diverse than in the other seasons, and two species of *Heterocapsa rotundata* (77.8 cells mL^−1^) and *Heterocapsa triquetra* (12.1 cells mL^−1^) were dominant. Similarly, the number of reads of *Heterocapsa triquetra* detected by the metagenomic analysis in the same sample were high. In Tongyeong, cell abundance of *Prorocentrum triestinum* (June) and *Cochlodinium polykrikoides* (September) was 341.1 and 2034 cells mL^−1^, respectively, which was similar to the metagenome result that the number of reads of *Prorocentrum* sp. and *Cochlodinium* sp. was 11,335, and 53,412, respectively. Small thecated dinoflagellate species, such as *Azadinium* sp. and *Bysmatrum* sp., occurred in the Seongsan region, located at Jeju Island. Some small nano-planktonic dinoflagellates, which are difficult to identify by microscopy, were easily found at Seongsan and Tongyeong using the metagenomic analysis (Table 2).

Although not all species of dinoflagellates identified by microscopic observation were included in the metagenomic analysis, the appearance of dominant species was found to be quite similar (Table 2).

### 3.5. Seasonal Distribution of Harmful Species Based on Metagenomic Analysis

**Figure 5:**
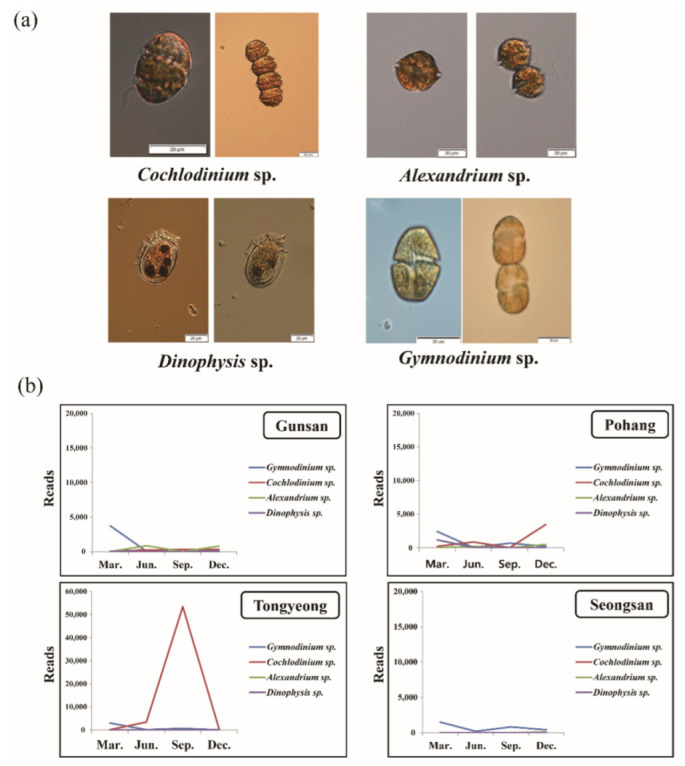
Seasonal distribution of red-tide-causing species through metagenome analysis. Photo of red-tide-causing species (*Cochlodinium* sp., *Alexandrium* sp., *Dinophysis* sp., *Gymnodinium* sp.) taken under a light microscope (**a**), seasonal changes in red-tide-causing species (**b**).

Four species of dinoflagellates (*Cochlodinium* sp., *Alexandrium* spp., *Dinophysis* spp., and *Gymnodinium* sp.) were selected as the causative species of red tide formation or toxin production in Korean waters (Figure 5a), and their seasonal distribution characteristics based on the number of reads through metagenomic analysis was confirmed by region. In Gunsan, the reads of *Gymnodinium* sp. were considerable in March, and *Dinophysis* spp. appeared in June and September. In Pohang, *Gymnodinium* sp. was relatively high in March, and *Cochlodinium* sp. was also detected at a high distribution in December. In Tongyeong, the abundance of *Cochlodinium* sp. was especially high in September, when a red tide from this species was occurring. In Seongsan, the appearance of *Gymnodinium* sp. in March and September was revealed by microscopic observation (Figure 5b). Based on these findings, the seasonal distribution of red tide-causing species, which was not confirmed by microscopic observation, was confirmed using metagenomic analysis.

## 4. Discussion

Approximately 300 dinoflagellate species are known to cause red tides and produce toxins worldwide, and these harmful events are increasing with changes in human activities and the environment [4]. Toxic dinoflagellate blooms frequently occur in the southern coastal waters of Korea, where many cage fish farms are located. As shown in Table 1, *Cochlodinium* sp. were dominant at Tongyeong in September according to NGS, which corresponds to the cell abundance counted by microscopic observation. In June, the NGS result that *Prorocentrum* sp. were mainly observed at Tongyeong was similar to the occurrence detected by microscopy analysis at this location. In addition, *Karlodinium* sp., which produces Karlotoxin and induces hemolytic and cytotoxic activity associated with fish mortality, appeared in our NGS results [27].

In a situation where the morphological analysis method is the dominant method for diagnosing harmful dinoflagellates off Korean coasts, diagnosis using molecular biology is considered to be a more objective number, and the development of technology through this method can lead to the development of new monitoring techniques [28]. Moreover, if NGS technology has been developed and applied to the monitoring of marine organisms, it is possible to simultaneously analyze a large amount of mixed samples and save the effort and time of long-term monitoring and research analysis [29,30,31].

Monitoring of marine microalgae using NGS has been used by many researchers because of its various advantages [11]. Metagenomic analysis using NGS has revealed a significant number of phytoplankton taxa previously missed by microscopy in recent efforts to sequence marine microorganisms [32]. Our study also revealed a significant number of dinoflagellate communities that could not be distinguished microscopically. The genetic analysis method used in this study, especially high-throughput sequencing, has shown effectiveness in the study of phytoplankton diversity and ecology, and it is considered that it can potentially replace the microscopic identification and population quantification methods currently used.

Light microscopy, which has been used for morphological classification and population evaluation, requires an extensive amount of consideration. Underestimation of phytoplankton, including dinoflagellates, in microscopic samples results in cell loss of taxa during preservation, storage, and handling, preferentially after treatment of samples with fixing fluid. Further, when counting cells, a sedimentation chamber is commonly used, which means that smaller cells that do not sink sufficiently are less counted or missed [33]. Moreover, identification of small dinoflagellates using microscopy is not easy when their cell size is under 20 µm with similar morphologies when fixed with Lugol’s solution [34]. We found that a significant number of dinoflagellate species were confirmed by metagenomic analysis compared to that by microscopic analysis. The small dinoflagellate cells which were classified as ‘small naked dinoflagellate’ were positively identified as species belonging to the genera *Amphidiniopsis*, *Biecheleria*, *Gymnodinium*, *Gyrodiniellum*, *Paragymnodinium*, *Pelagodinium*, and *Symbidinium*, while ‘small thecated dinoflagellate’ included *Apicoporus*, *Azadinium*, *Crypthecodinium*, *Durinskia*, *Heterocapsa*, and *Pfiesteria*. In particular, the sand-dwelling dinoflagellate *Bysmatrum arenicola*, which is easily confused with *Scrippsiella* [35] in microscopic analysis, was found in the metagenomic analysis in June at Seongsan (Figure 3d). This suggests that the metagenomic analysis was more extensive.

Although the NGS technique showed a high resolution for species identification compared to that with conventional microscopic analysis, further studies are required for development of an understanding of the spatial and seasonal dynamics of the dinoflagellate community using NGS-based metagenomics. Thus far, molecular markers based on ribosomal DNA have usually been used to identify the species, even among relatives [36]. However, this approach is limited by interspecific divergence, while it is difficult to distinguish intraspecific variation. As the reference database of dinoflagellates via the NGS method in this study was established based on 18S rDNA sequences, the relative proportions of some dinoflagellates in field samples could be misidentified in the presence of other dinoflagellates which were similar. Large subunit (LSU) rDNA sequences of *Prorocentrum* species containing *P. rhathymum*, *P. mexicanum*, and *P.* cf. *rhathymum*, which are toxic, were closer to the relatives, showing 0.1–0.9% dissimilarity, and small subunit rDNA (SSU) sequences of most of these are nearly identical [37]. Edvardsen et al. [38] reported that SSU rDNA sequences among *Dinophysis acuminata*, *Dinophysis acuta*, and *Dinophysis norvegica* show approximately 0.3% distance, and differences of LSU rDNA sequences among these species show 0.4–1.6% distance. Moreover, species whose sequences are not available in the GenBank are hardly detected despite their potential presence in the sample analyzed by the NGS technique because of the absence of deposited sequences. To distinguish intraspecific similarity of the above-mentioned species, establishment of a reference database via the NGS technique based on biomarkers such as cytochrome c oxidase I (COX1) and the cytochrome b (COB) gene which allows for the unambiguous identification of the species should be developed.

Metagenomic analysis of marine biodiversity and abundance based on NGS will provide precise indicators for understanding biological patterns and characteristics of species in different habitats. Given the lack of molecular reference library databases, it is necessary to collect vast amounts of sequence information targeting biomarkers such as SSU, LSU, COX1, and COB genes. However, in this study, we established a reference database of dinoflagellates that occur in the coastal waters of Korea based on SSU rDNA sequences using the NGS technique and analyzed field samples in the presence of this NGS reference database library. We expect that the newly established reference database via the NGS will provide a better understanding of the seasonal dynamics of toxic dinoflagellates, as well as a complementary approach to conventional microscopic analysis for monitoring dinoflagellate community compositions.

## 5. Conclusions

This study integrated analyses of high-resolution dinoflagellate community composition and distribution in South Korea. Altogether, the results presented here reveal a complex dinoflagellate community pattern. The NGS-based (18S rRNA amplicon) metagenomics were able to detect dinoflagellates with low abundance, and allow continuous monitoring of the phytoplankton community in environmental samples even though numerous DNA samples were simultaneously collected compared to the conventional microscopic analysis. Our analysis suggested that NGS-based characterization of the 18S rRNA gene holds great promise as a tool for phytoplankton monitoring, as it allows for simultaneous regional cluster analysis monitoring in a high-throughput, reproducible, and cost-effective manner.

In today’s world, which requires advances in environmental monitoring due to large-scale blooming of toxic algae and international regulations regarding their toxic substances, this study provides a technique for the rapid evaluation of environmental samples for existing taxa of major dinoflagellates and potentially harmful/invasive species. In addition, the extension of the reference database presented in this study and addition of the species list can further expand the taxonomic scope so it can be applied to real-time monitoring of temporal dynamics and species diversity problems of harmful algal blooms in a wide range of waters.

## Figures and Tables

**Table 1:** Seasonal variations and distribution of dinoflagellates in four coastal waters (Gunsan, Pohang, Tongyeong, and Seongsan) by metagenomic analysis. Total dinoflagellate reads and unidentified reads (**a**), and proportion(%) of the 10 most common dinoflagellate species (**b**).

(a)								
Location	March		June		September		December	
	Dinoflagellate Reads	Unidentified	Dinoflagellate Reads	Unidentified	Dinoflagellate Reads	Unidentified	Dinoflagellate Reads	Unidentified
Gunsan	47,588	16,263	36,787	3084	39,780	14,265	87,273	11,245
Pohang	34,641	16,445	76,439	12,304	7222	4621	75,121	14,332
Tongyeong	12,397	3606	22,549	2855	69,468	1425	69,819	14,224
Seongsan	8924	2060	60,769	8994	30,197	4962	42,927	8644
**(b)**								
**Location**	**March**		**June**		**September**		**December**	
	**Proportion (%)**	**Species**	**Proportion (%)**	**Species**	**Proportion (%)**	**Species**	**Proportion (%)**	**Species**
Gunsan	32.1	*Karlodinium veneficum*	45.2	*Gonyaulax* sp.	24.1	*Karlodinium* sp.	34.2	*Gyrodinium* sp.
	24.2	*Gyrodinium* sp.	15.3	*Symbiodinium* sp.	17.6	*Akashiwo* sp.	24.8	*Amphidiniella* sp.
	7.9	*Gymnodinium* sp.	7.9	*Karlodinium veneficum*	5.5	*Sinophysis* sp.	10.5	*Ceratium* sp.
	0.4	*Noctiluca scintillans*	6.3	*Ceratium* sp.	4.2	*Peridinium* sp.	4.5	*Heterocapsa triquetra*
	0.3	*Symbiodinium* sp.	3.1	*Pelagodinium* sp.	2.9	*Scrippsiella trochoidea*	2.5	*Karlodinium* sp.
	0.3	*Protoperidinium* sp.	2.4	*Dissodinium pseudolunula*	2.5	*Katodinium* sp.	2.4	*Peridinium* sp.
	0.3	*Pelagodinium* sp.	2.4	*Alexandrium* sp.	1.7	*Pelagodinium* sp.	2.1	*Noctiluca scintillans*
	0.2	*Scrippsiella* sp.	1.7	*Gyrodinium* sp.	1.3	*Gyrodinium* sp.	1.3	*Katodinium* sp.
	0.2	*Dinophysis* sp.	1.2	*Azadinium* sp.	0.8	*Cochlodinium* sp.	0.9	*Akashiwo* sp.
	0.2	*Ceratium* sp.	1.1	*Amphidiniopsis* sp.	0.5	*Ceratium* sp.	0.7	*Gonyaulax* sp.
Pohang	19.0	*Katodinium* sp.	52.1	*Gyrodinium* sp.	16.0	*Karlodinium* sp.	27.2	*Gyrodinium* sp.
	10.1	*Gyrodinium* sp.	4.9	*Heterocapsa triquetra*	6.8	*Sinophysis* sp.	16.2	*Bysmatrum arenicola*
	6.9	*Gymnodinium* sp.	4.0	*Ceratium* sp.	4.1	*Akashiwo* sp.	7.7	*Karlodinium veneficum*
	5.4	*Azadinium* sp.	3.7	*Karlodinium* sp.	1.5	*Paragymnodinium* sp.	7.4	*Akashiwo* sp.
	3.3	*Dinophysis* sp.	2.8	*Heterocapsa circularisquama*	1.5	*Peridinium* sp.	7.3	*Ceratium* sp.
	1.5	*Pelagodinium* sp.	2.1	*Gonyaulax* sp.	1.0	*Amphidiniella* sp.	4.6	*Cochlodinium* sp.
	1.4	*Ceratium* sp.	1.9	*Pelagodinium* sp.	1.0	*Ceratium* sp.	2.9	*Azadinium* sp.
	0.9	*Gonyaulax spinifera*	1.1	*Prorocentrum* sp.	0.5	*Bysmatrum arenicola*	1.5	*Katodinium* sp.
	0.9	*Gonyaulax* sp.	0.9	*Peridinium* sp.	0.4	*Pelagodinium* sp.	0.6	*Alexandrium* sp.
	0.8	*Erythropsidinium* sp.	0.6	*Cochlodinium* sp.	0.4	*Scrippsiella trochoidea*	0.6	*Peridinium* sp.
Tong-yeong	23.9	*Gyrodinium* sp.	50.2	*Prorocentrum* sp.	77.3	*Cochlodinium* sp.	62.3	*Gyrodinium* sp.
	23.6	*Gymnodinium* sp.	5.9	*Gyrodinium* sp.	7.9	*Gyrodinium* sp.	7.5	*Symbiodinium* sp.
	19.7	*Karlodinium veneficum*	5.6	*Scrippsiella* sp.	5.0	*Noctiluca scintillans*	1.5	*Noctiluca scintillans*
	0.9	*Cochlodinium* sp.	5.3	*Karlodinium* sp.	3.7	*Bysmatrum arenicola*	0.6	*Karlodinium* sp.
	0.9	*Pelagodinium* sp.	3.8	*Noctiluca* sp.	1.7	*Protoperidinium* sp.	0.6	*Alexandrium* sp.
	0.6	*Noctiluca scintillans*	3.5	*Neoceratium* sp.	0.9	*Karlodinium* sp.	0.6	*Peridinium* sp.
	0.3	*Akashiwo* sp.	1.3	*Heterocapsa* sp.	0.5	*Ceratium* sp.	0.6	*Amphidiniopsis* sp.
	0.2	*Paragymnodinium* sp.	1.1	*Blastodinium* sp.	0.4	*Erythropsidinium* sp.	0.5	*Heterocapsa triquetra*
	0.2	*Pfiesteria piscicida*	1.1	*Protodinium* sp.	0.4	*Akashiwo* sp.	0.4	*Cochlodinium* sp.
			1.0	*Chytriodinium* sp.	0.3	*Heterocapsa triquetra*	0.2	*Scrippsiella trochoidea*
Seong-san	29.1	*Gyrodinium* sp.	56.1	*Bysmatrum arenicola*	19.2	*Karlodinium veneficum*	22.4	*Gyrodinium* sp.
	16.6	*Gymnodinium* sp.	13.2	*Gyrodinium* sp.	11.1	*Bysmatrum arenicola*	19.3	*Karlodinium* sp.
	8.4	*Erythropsidinium* sp.	7.4	*Erythropsidinium* sp.	11.0	*Gyrodinium* sp.	13.9	*Bysmatrum arenicola*
	3.9	*Karlodinium* sp.	5.6	*Karlodinium* sp.	10.4	*Ceratium* sp.	6.8	*Ceratium* sp.
	1.7	*Heterocapsa* sp.	1.6	*Ceratium* sp.	8.2	*Peridinium* sp.	5.5	*Akashiwo* sp.
	0.7	*Paragymnodinium* sp.	1.3	*Pelagodinium* sp.	1217	*Akashiwo* sp.	2.3	*Heterocapsa* sp.
	0.3	*Azadinium* sp.	1.2	*Heterocapsa triquetra*	4.9	*Peridiniopsis* sp.	1.7	*Peridinium* sp.
	0.3	*Akashiwo* sp.	0.9	*Azadinium* sp.	4.0	*Gymnodinium catenatum*	1.5	*Azadinium* sp.
	0.2	*Cochlodinium* sp.	0.4	*Akashiwo* sp.	3.7	*Azadinium* sp.	1.1	*Noctiluca scintillans*
	0.2	*Pelagodinium* sp.	0.4	*Symbiodinium* sp.	2.6	*Heterocapsa circularisquama*	1.0	*Peridiniopsis* sp.

**Table 2:** Species composition and cell number of dinoflagellates analyzed by microscopic observation. Seasonal (March, June, September, December) species composition in four coastal regions (Gunsan, Pohang, Tongyeong, Seongsan).

Location	March		June		September		December	
	Cell/mL	Species	Cell/mL	Species	Cell/mL	Species	Cell/mL	Species
Gunsan	2.7	*Heterocapsa triquetra*	2.7	*Scrippsiella* sp.	0.8	*Gyrodinium* sp.	2.9	*Gymnodinium* sp.
	0.9	*Gyrodinium* sp.	2.5	*Ceratium fusus*	0.1	*Peridiniopsis* sp.	2.9	*Gyrodinium* sp.
	0.9	*Prorocentrum micans*	2.5	*Heterocapsa rotundata*	0.1	*Protoperidinium divergence*		
	0.9	*Pyrocystis lunula*	1.8	*Gonyaulax* sp.				
			1.2	*Prorocentrum* sp.				
			0.9	*Dissodinium pseudolunula*				
			0.6	*Ceratium* sp.				
			0.6	*Ceratium tripos*				
			0.6	*Karlodinium* sp.				
			0.6	*Prorocentrum micans*				
Pohang	3.6	*Gymnodinium* sp.	77.8	*Heterocapsa rotundata*	1.7	*Heterocapsa rotundata*	0.4	*Gymnodinium* sp.
	1.8	*Gyrodinium* sp.	12.1	*Heterocapsa triquetra*	1.7	*Scrippsiella* sp.		
	1.4	*Ceratium kofoidii*	7.8	*Gymnodinium* sp.	0.8	*Prorocentrum triestinum*		
	1.2	*Alexandrium* sp.	4.3	*Protopeidinium pyriforme*	0.8	*Gymnodinium* sp.		
	0.5	*Heterocapsa rotundata*	2.6	*Gyrodinium* sp.	0.8	*Gyrodinium* sp.		
			2.6	*Ceratium kofoidii*				
			1.7	*Alexandrium* sp.				
			0.9	*Amphidinium operculatum*				
Tongyeong	1.6	*Small thecated dinoflagellate	341.1	*Prorocentrum triestinum*	2034	*Cochlodinium polykrikoides*	1.6	*Gymnodinium* sp.
	0.7	*Small naked dinoflagellate	18.0	*Small naked dinoflagellate	28.8	*Karlodinium* sp.		
	0.1	*Alexandrium* sp.	17.0	*Small thecated dinoflagellate	18.0	*Gyrodinium* sp.		
	0.1	*Gymnodinium* sp.	14.9	*Scrippsiella* sp.	5.4	*Prorocentrum* sp.		
	0.1	*Karlodinium* sp.	11.7	*Peridinium* sp.	3.6	*Bysmatrum* sp.		
			10.6	*Alexandrium* sp.	3.6	*Ceratium* sp.		
			3.2	*Heterocapsa* sp.	1.8	*Alexandrium* sp.		
			3.2	*Scrippsiella trochoidea*	1.8	*Heterocapsa* sp.		
			2.1	*Protoperidinium* sp.				
			1.1	*Gonyaulax* sp.				
			1.1	*Gymnodinium* sp.				
Seongsan	0.6	*Small naked dinoflagellate	5.8	*Azadinium* sp.	0.6	*Small naked dinoflagellate	1.3	*Bysmatrum* sp.
	0.2	*Bysmatrum* sp.	5.8	*Bysmatrum* sp.	0.5	*Peridiniopsis* sp.	1.0	*Gymnodinium* sp.
	0.2	*Katodinium* sp.	5.4	*Small naked dinoflagellate	0.3	*Gymnodinium* sp.	0.3	*Gyrodinium* sp.
	0.2	*Prorocentrum* sp.	2.4	*Small thecated dinoflagellate	0.3	*Prorocentrum minimum*		
			1.4	*Gymnodinium* sp.				
			1.0	*Protoperidinium pellucidum*				
			0.3	*Heterocapsa* sp.				
			0.3	*Peridiniopsis* sp.				
			0.3	*Prorocentrum* sp.				
			0.3	*Protoperidinium* sp.				
			0.3	*Woloszynskia* sp.				

*Small thecated dinoflagellates: *Apicoporus*, *Azadinium*, *Crypthecodinium*, *Durinskia*, *Heterocapsa*, *Pfiesteria*. *Small naked dinoflagellates: *Amphidiniopsis*, *Biecheleria*, *Karlodinium*, *Gymnodinium*, *Gyrodiniellum*, *Paragymnodinium*, *Pelagodinium*, *Symbidinium*.

## Data Availability

Not applicable.

## Supplementary Materials

The following supporting information can be downloaded at: https://www.mdpi.com/article/10.3390/microorganisms10071459/s1, Figure S1: Workflow chart for metagenome analysis, Using CLC Genomics Workbench program. Supplementary Table S1.
