# Supplementary figures and images for: Metagenomic Analysis of the Species Composition and Seasonal Distribution of Marine Dinoflagellate Communities in Four Korean Coastal Regions
Source: Microorganisms. 2022 Jul 19;10(7):1459. doi: 10.3390/microorganisms10071459 (PMC9320301; doi:10.3390/microorganisms10071459)

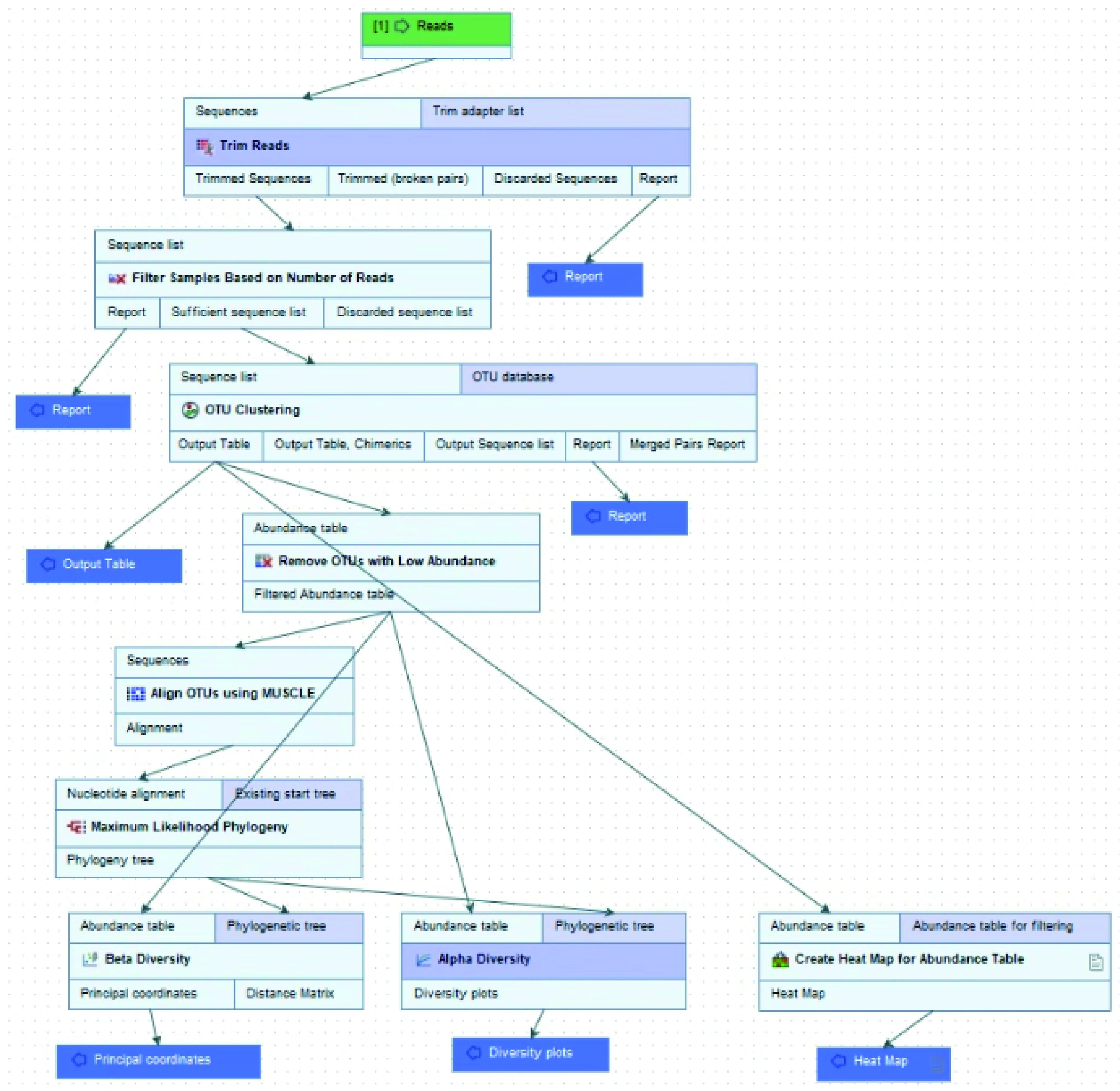

Supplement: Supplementary file 1 [file microorganisms-10-01459-s001.zip › microorganisms-1726911-supplementary.tif]
